# Supplementary material for: Improved Functionality of Integration-Deficient Lentiviral Vectors (IDLVs) by the Inclusion of IS2 Protein Docks
Source: Pharmaceutics. 2021 Aug 6;13(8):1217. doi: 10.3390/pharmaceutics13081217 (PMC8401568; doi:10.3390/pharmaceutics13081217)
Supplement: Supplementary file 1 [file pharmaceutics-13-01217-s001.zip › pharmaceutics-1314233-SI.pdf]

pharmaceutics

Supplementary Materials: Improved Functionality of Integration-Deficient Lentiviral Vectors (IDLVs) by the Inclusion of IS2 Protein Docks

Marina Cortijo-Gutierrez, Sabina Sánchez-Hernández, María Tristán-Manzano, Noelia Maldonado-Pérez, Lourdes Lopez-Onieva, Pedro J. Real, Concha Herrera, Juan Antonio Marchal, Francisco Martin and Karim Benabdellah

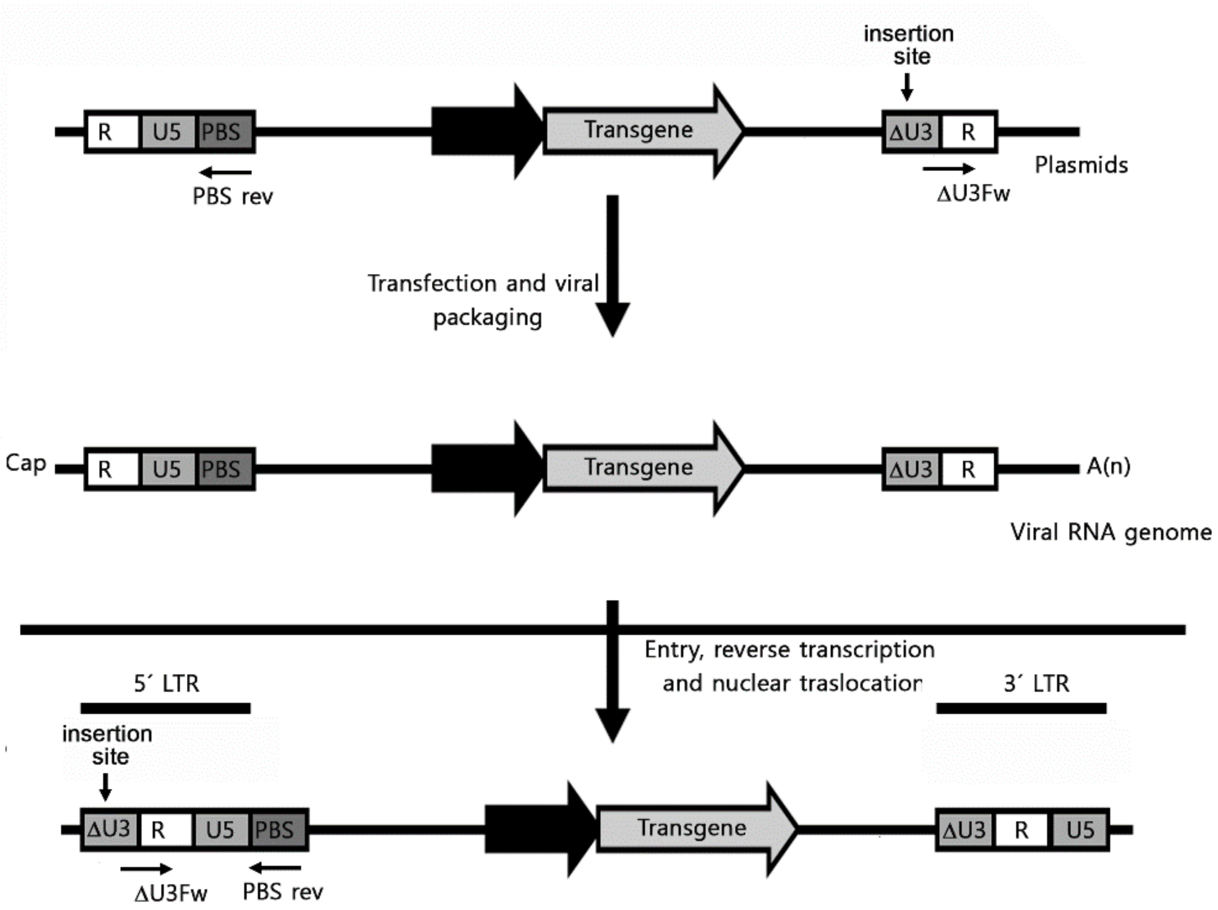

Primer pair	Sequence	Amplification
ΔU3Fw	5´GATCTGCTTTTTGCTTGTACT 3´	Viral genome within the cells
PBS rev	5´GAGTCCTGCGTCGAGAGAAGC 3´	
hAlbFw	5´GCTGTAATCTCTTGTGGGCTGT 3´	Genomic normalization
hAlbrev	5´ACTCATGGGAGCTGCTGGTTC 3´	

Figure S1. Scheme of viral reverse transcription process and oligonucleotide primers used for the specific amplification of the viral genome within the transduced cells to estimate the amount of viral DNA genomes. The primers were designed in order to discriminate between the plasmid used for the transfection and the production of the lentiviral vector and the final viral genome within the cells, thus drastically reducing false positives. Please note that the qPCR primers were designed to obtain the same amplicon size in both IDLV variants, thus ruling out any decrease in PCR efficiency due to IS2 insertion.

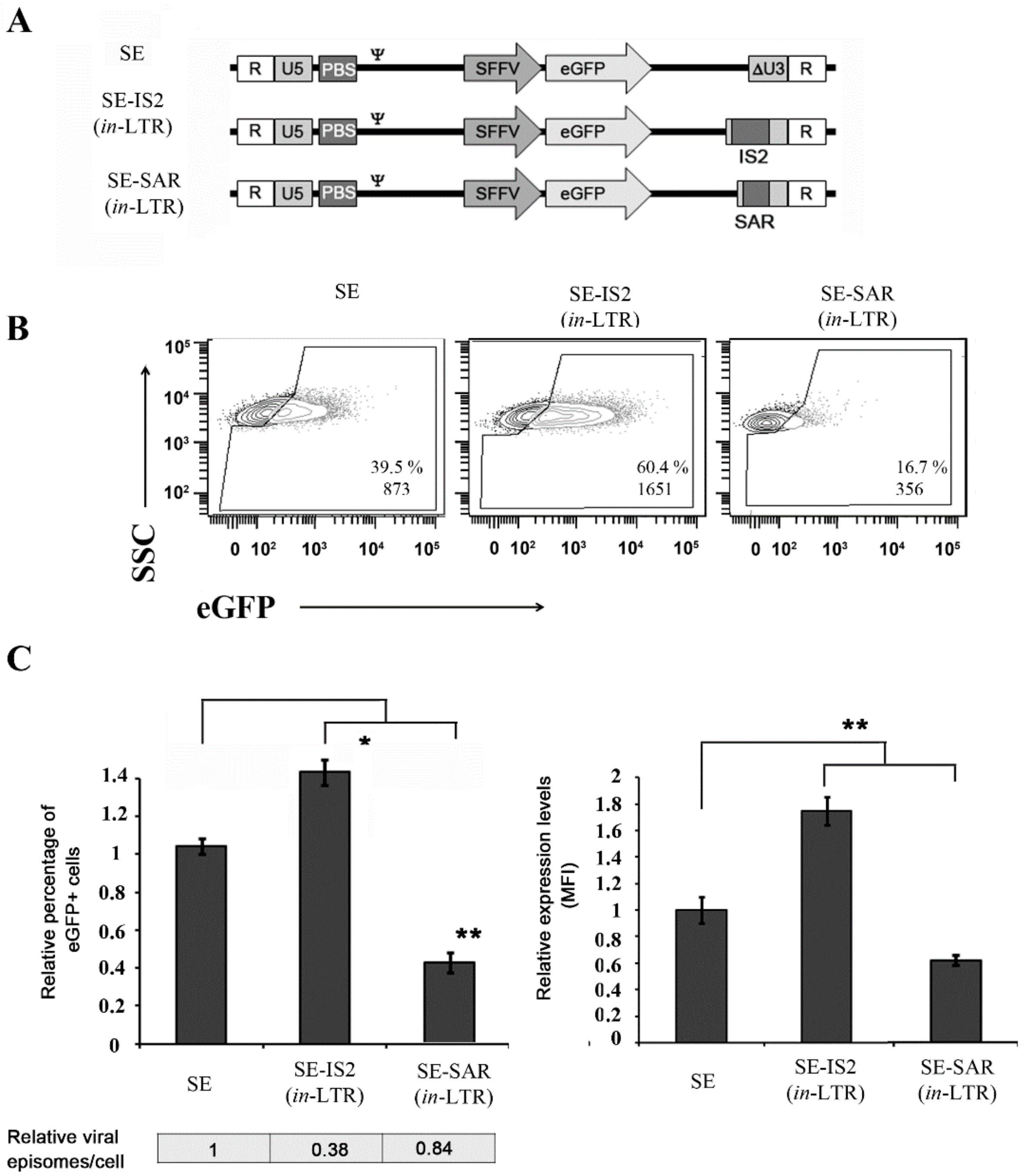

Figure S2. Comparison of the effects of IS2 and SAR elements on 293T cells in the LTR. **A**. Schematic diagram of the IS2 and SAR based IDLVs. Two different elements were inserted into the U3 region of the 3′LTR (see Materials and Methods for details). **B.** Representative plots of 293T cells transduced with SE, SE-IS2 (*in*-LTR) and SE-SAR (*in*-LTR) which were analysed 72 h after transduction. **C.** Effect of inclusion of IS2 or SAR on the LTR. The graph represents the percentage (bottom left) of eGFP+ cells transduced and mean fluorescence intensity (MFI) (bottom right) of eGFP+ cells transduced with SE-IS2 (*in*-LTR) or SE-SAR (*in*-LTR) relative to SEs. The graph represents the mean of at least three independent experiments, while the error bar indicates the standard error of the mean (SEM); *=p<0.01; **=p<0.05; two-tailed unpaired Student's t-test.

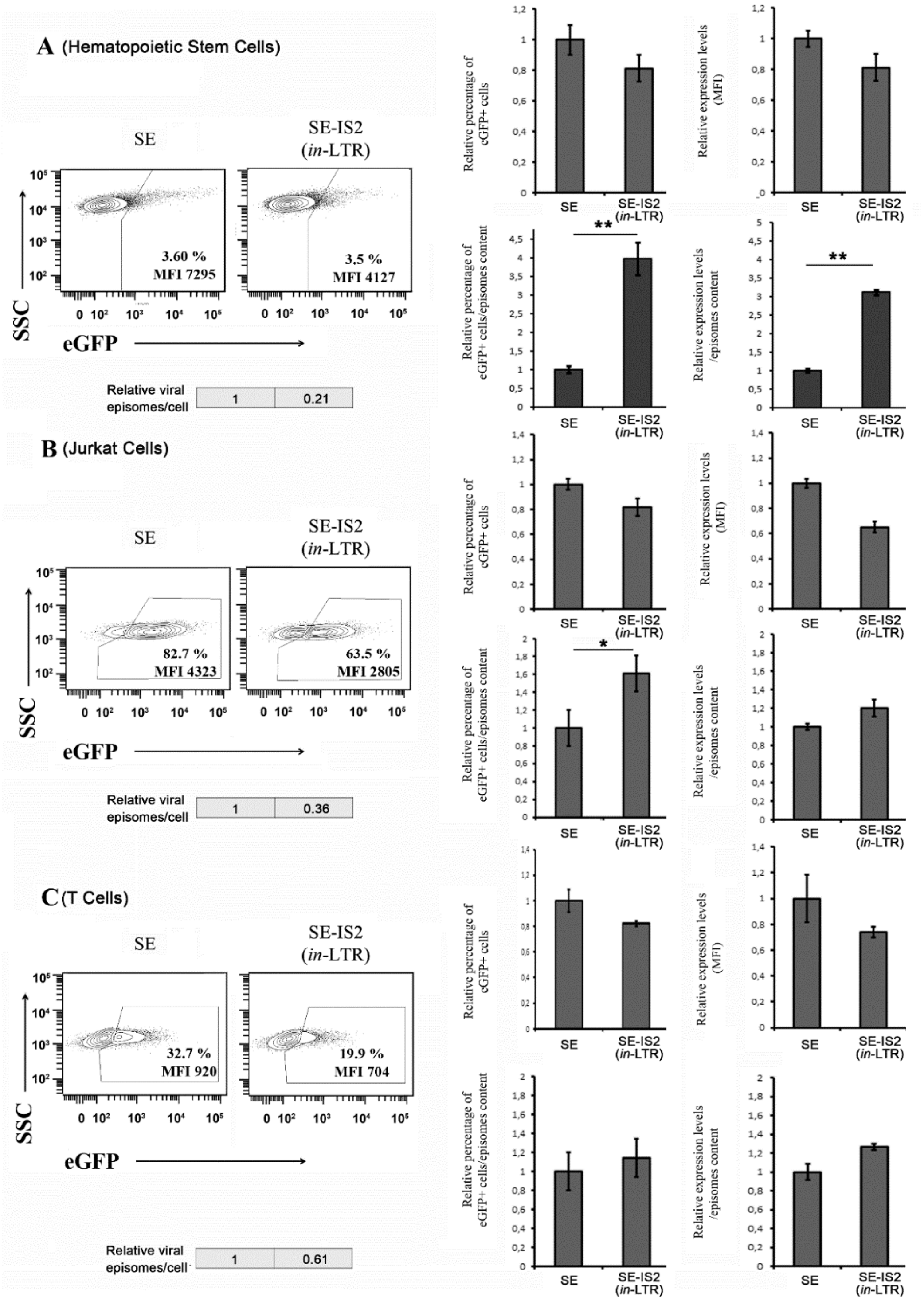

Figure S3. Comparison of the effects of IS2 on different cells types. **A.** On the left, representative plots of human hematopoietic stem cells (hHSCs) transduced with SE and SE-IS2 (*in*-LTR) IDLVs which were analysed 72 h post-transduction. Graphs on the right show the percentage of eGFP+ cells transduced and mean fluorescence intensity (MFI) of eGFP+ cells transduced relative to SE. Bottom right: graphs showing the estimated efficiency of the different constructs (as detailed in Materials and Methods). **B** and **C** show the same data, but related to Jurkat and primary T cells, respectively, transduced with SE and SE-IS2 (*in*-LTR) IDLVs. All graphs represent mean of at least three separate experiments, while error bars indicate standard error of the mean (SEM); **=p<0.001; *=p<0.03; two-tailed unpaired Student's t-test.

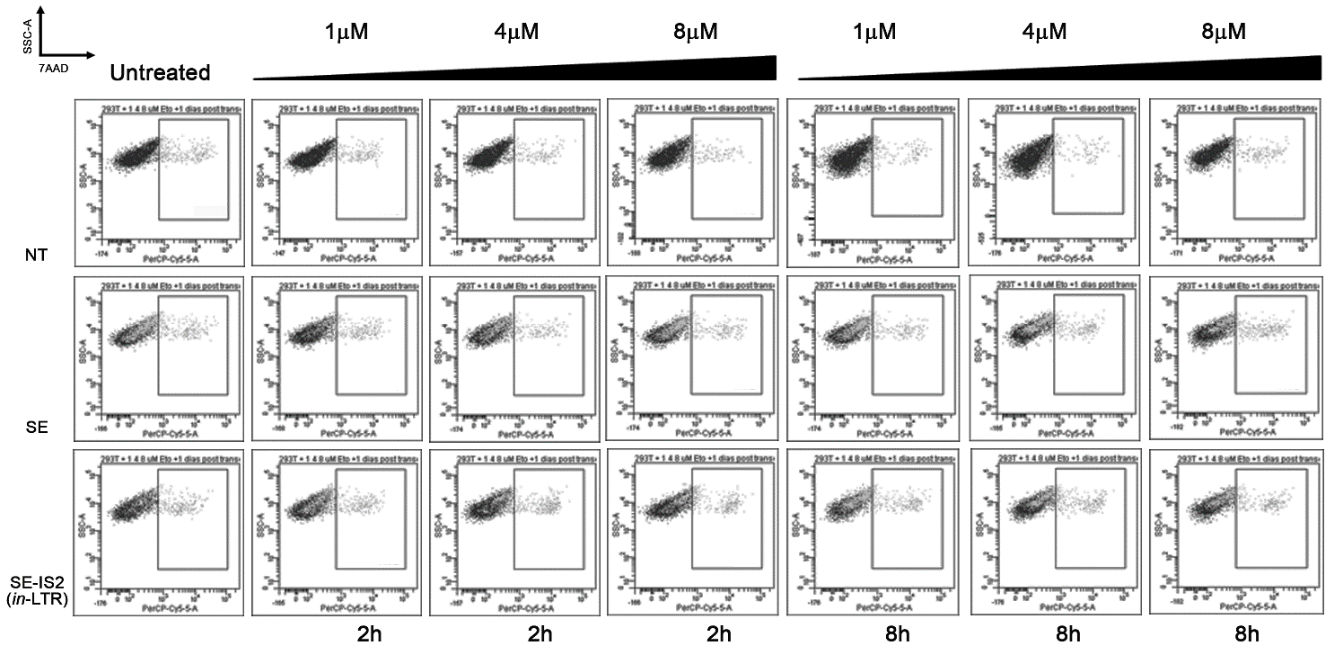

Figure S4. Description of target cell apoptosis induced by treatment with etoposide. Untreated cells (NT) and cells treated with 1, 4 or 8 mM etoposide for 2 or 8 hours were stained with 7 amino-actinomycin D (7-AAD) for apoptosis estimation. The duration and strength of the etoposide treatment did not induce a significative increase in the apoptotic population.

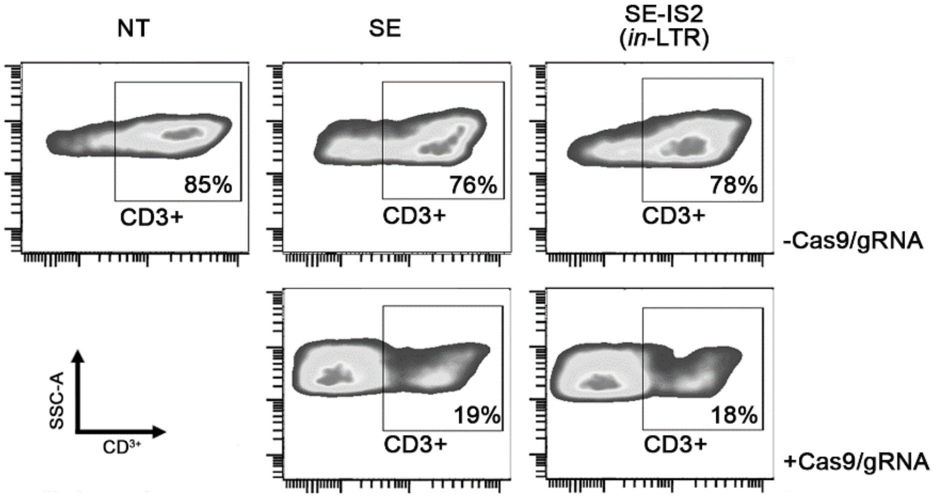

Figure S5. RNP nucleofection leads to highly efficient gene editing in Jurkat cells. CD3+ expression analyzed by flow cytometry 72 hours after RNP nucleofection using gRNA to target the exon1 of the constant chain of the TCR locus.
